# Calculating fluoride concentrations data using ambient temperatures in drinking water distribution networks in select provinces of Iran

**DOI:** 10.1016/j.dib.2017.08.054

**Published:** 2017-09-05

**Authors:** Mohammad Ali Zazouli, Reza Sadeghnezhad, Laleh R. Kalankesh

**Affiliations:** a Department of Environmental Health Engineering, Health Sciences Research Center, Faculty of Health, Mazandaran University of Medical Sciences, Sari, Iran; b Department of Environmental Health Engineering, Health Sciences Research Center, Student Research Committee, Mazandaran University of Medical Sciences, Sari, Iran; c Department Environmental Health Engineering, Ph.D. Candidate of Health science Research Center, Student Research Committee Mazandaran University of Medical Sciences, Sari, Iran

**Keywords:** Fluoride, Drinking water supplies, Ambient temperature, Iran

## Abstract

Fluoride concentrations in drinking water were analyzed relative to air temperature data collected in different provinces of Iran. Determining suitable concentrations of fluoride in drinking water is crucial for communities because of the health effects of fluoride on humans. This study analyzed fluoride concentrations in drinking water from selected Iranian provinces. The data were derived mainly from a detailed literature review. The annual mean maximum temperatures (AMMTs) were collected from a popular website that maintains records of daily ambient temperature measurements for the last five years (2012–2016). Using regional ambient temperatures, the optimal value of fluoride in drinking water for each province was calculated by the Galgan and Vermillion formula. These optimal fluoride concentrations in drinking water for different Iranian regions were calculated to be 0.64–1.04 mg F/L. Most of the selected provinces were found to have acceptable concentrations of fluoride, except for Alborz, Khuzestan, and Hormozgan, which reported concentrations of 0.66, 0.66, and 0.64 mg/L, respectively.

**Specifications Table**

**Table:** 

**Subject area**	Environmental health
**More specific subject area**	Concentrations of fluoride in drinking water impacting daily fluoride intake
**Type of data**	Fluoride concentrations in drinking water
**How data was acquired**	First data was acquired through a literature search and from a local meteorological organization
**Data type**	Raw and analyzed data
**Experimental factors**	The relevant data were collected using keywords including “drinking water,” “fluoride,” “fluoride concentration,” and “temperature.” Monthly maximum ambient temperatures for selected provinces in Iran were obtained from the website of the world Meteorological Organization (WMO). WMO is a specialized agency of the United Nations. It is the UN system's authoritative voice on the state and behavior of the Earth's atmosphere
**Experimental features**	The optimal amount of fluoride in drinking water was calculated using the local temperature and the Galgan and Vermillion formula.
**Data source location**	Iran
**Data accessibility**	The relevant data are reported in this article.

**Value of the data**

• The collected data and the Galgan and Vermillion formula were used to calculate the fluoride concentrations in drinking water for selected Iranian provinces.
• The results identify provinces that have critical fluoride concentrations in drinking water.
• Sharing such data can enable much earlier rectification of the issue and therefore lessen the possible negative impacts arising from consumption of polluted water.
• Combining the reported data on fluoride concentrations in drinking water with information on ambient temperature is very useful; this study is the first to attempt this methodology successfully in the Iranian context.

## 1 Data

Table 1, Table 2 show data obtained through the literature review and calculated using the method described. Table 1 shows fluoride concentration in water supplies in different provinces of Iran. Table 2 lists the AAMT data by provinces and the calculated optimal fluoride concentrations in their respective water supply systems.

**Table 1:** Fluoride concentrations (mg/L) in drinking water supplies of selected provinces of Iran.

**Name of province**	**Minimum**	**Average**	**Maximum**	**References**
**Alborz**	0	0.32	0.72	[1]
**Ardabil**	0.12	0.32	0.58	[2]
**Azerbaijan, East**	0.19	0.343	0.847	[3]
**Azerbaijan, West**	a	0.40	a	[4]
**Bushehr**	0.7	0.48	0.48	[5]
**Chaharmahal and Bakhtiari**	0.7	0.14	0.46	[6]
**Fars**	0.47	0.69	1.26	[7]
**Gilan**	<0.02	0.219	0.82	[2]
**Golestan**	0.7	0.45	0.45	[8]
**Hamadan**	a	0.57	1.78	[9]
**Hormozgan**	a	0.74	a	[10]
**Ilam**	0.18	0.42	0.59	[11]
**Isfahan**	<0.029	1.5	0.292	[2]
**Kerman**	0.04	0.17	0.27	[12]
**Kermanshah**	0.01	0.193	0.86	[2]
**Khorasan, North**	a	a	0.78	[13]
**Khorasan, Razavi**	0.11	0.88	3.06	[14]
**Khorasan, South**	a	a	0.52	[15]
**Khuzestan**	a	a	0.26	[16]
**Kohgiluyeh and Boyer-Ahmad**	0.81	0.01	0.264	[2]
**Kurdistan**	0.01	0.31	0.59	[17]
**Lorestan**	a	a	0.70	[18]
**Markazi**	a	0.48	a	[19]
**Mazandaran**	0.17	0.24	0.31	[20]
**Qazvin**	0.18	0.662	1.8	[2]
**Qom**	0.21	0.82	1.28	[21]
**Semnan**	0.13	0.742	1.49	[2]
**Sistan and Baluchestan**	0.1	0.93	2.1	[22]
**Tehran**	0.40	0.7	2.1	[23]
**Yazd**	<0.02	0.5	1.39	[2]
**Zanjan**	0.26	0.03	0.03	[24]

a Fluoride concentrations were not reported in these publications.

**Table 2:** Calculated optimal fluoride concentrations (mg/L) in the drinking water supplies, using the Galgan and Vermillion formula and annual mean maximum temperature (AMMT) data).

**Name of province**	**AAMT (°C)**	**F**^−^**(mg/L)**	**Name of province**	**AAMT (°C)**	**F**^−^**(mg/L)**
**Alborz**	32.66	0.66	**Khorasan, Razavi**	21.38	0.85
**Ardabil**	14.88	1.04	**Khorasan, South**	23.88	0.79
**Azerbaijan, East**	18.33	0.93	**Khuzestan**	32.66	0.64
**Azerbaijan, West**	18.38	0.93	**Kohgiluyeh and Boyer-Ahmad**	22.3	0.83
**Bushehr**	27.55	0.72	**Kurdistan**	15.05	1.03
**Chaharmahal and Bakhtiari**	23	0.81	**Lorestan**	20.22	0.88
**Fars**	25.66	0.76	**Markazi**	20.22	0.88
**Gilan**	21.4	0.85	**Mazandaran**	23.05	0.81
**Golestan**	20.38	0.87	**Qazvin**	22.4	0.82
**Hamadan**	20.22	0.88	**Qom**	25.5	0.76
**Hormozgān**	31.66	0.66	**Semnan**	25.2	0.77
**Ilam**	23.05	0.81	**Sistan and Baluchestan**	16.88	0.97
**Isfahan**	20	0.88	**Tehran**	23	0.81
**Kerman**	24.7	0.78	**Yazd**	24.8	0.78
**Kermanshah**	22.5	0.84	**Zanjan**	20.7	0.86
**Khorasan, North**	16.83	0.97			

The results of Table 1 show that the reported fluoride concentrations of most provinces are less than the calculated values reported in Table 2. However, some provinces, such as, Chaharmahal and Bakhtiari, Qom, Hormozgān, Isfahan, and Khorasan, Razavi, have fluoride concentrations higher than values calculated using the standard formula and AMMT data.

**Fig. 1:**
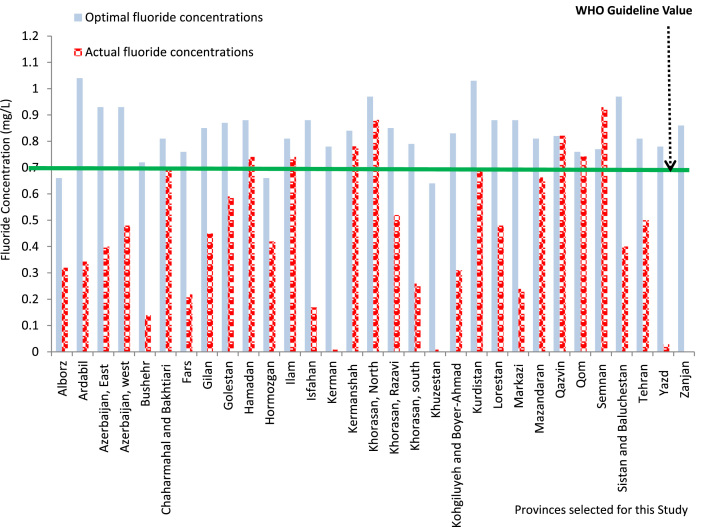
Comparison of calculated fluoride concentrations and values reported in the literature with the WHO guideline.

**Fig. 2:**
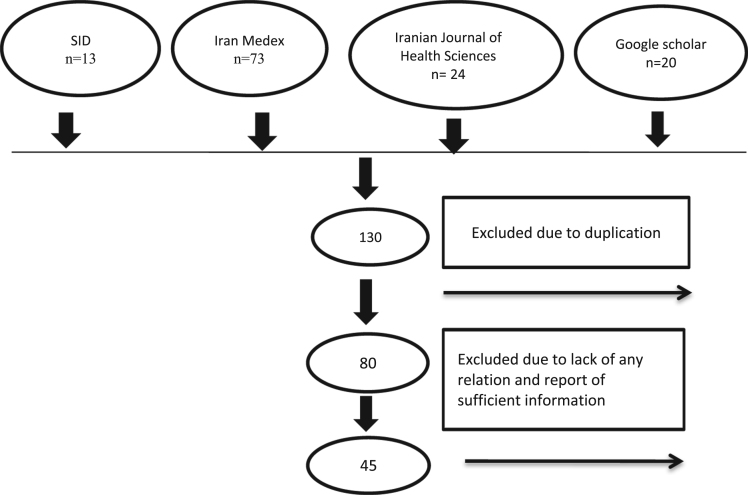
Flowchart showing the data selection process during the literature review.

Fig. 1 shows the comparison of the calculated fluoride concentrations in drinking water for various provinces of Iran as well as the values reported in the literature against the allowable concentration level according to the WHO guideline [25]. The minimum allowable concentration of fluoride (0.7 mg/L) is represented by the green line in Fig. 2, which also reveals that most of the selected provinces meet the stipulated guideline, except for Alborz, Khuzestan, and Hormozgan. The fluoride concentrations for these provinces were found to be less than 0.7 mg/L.

## 2 Experimental design, materials and methods

In this study, fluoride concentrations in drinking water and ambient temperatures for selected Iranian provinces were obtained through a literature search and publically published data. The publications used in the literature search mainly included Pub Med, Science Direct, Iran Medex, and SID from 1990 to 2016, as well as original research articles that reported fluoride concentrations in drinking water. Monthly maximum ambient temperature data were then obtained for the selected provinces from a popular website that provides records of ambient air temperatures (www.worldweatheronline.com). Published concentrations of fluoride in drinking water were found for 31 provinces of Iran (Fig. 2).

Articles published in both Persian and English languages were used in this research (Fig. 2).

Data categorization and analysis of subgroups were carried out to decrease the impact of confounding factors such as consumption of fluoride-containing supplements that can affect the fluoride concentrations in drinking water [11]. According to epidemiologists, ambient temperature is considered to be the most significant factor affecting fluoride concentration in drinking water. Therefore, categorization was based firstly on the province being studied, and secondly, on the fluoride concentration in drinking water. According to other studies, factors such as exposure time to fluoride in drinking water and any exposure to fluoride are note relevant to this study, and hence, these factors were not considered.

## 3 Calculation of optimal fluoride concentrations

The optimum fluoride concentration for a community may be determined simply by obtaining the mean maximum temperature for a 5-year or longer period. The recommended formula for determining fluoride concentrations in water relative to ambient temperatures was developed by Galgan and Vermillion (1957). It was determined that average maximum temperatures can influence fluoride concentrations in water supplies; therefore, annual mean maximum temperatures of various regions were used to calculate the optimal amount of fluoride in drinking or “potable” water [6].

$$\mathrm{Optimal}\mathrm{Fluoride}\mathrm{Concentration}(\frac{\mathrm{mg}}{L})=\frac{0.022}{0.0104+0.000724\times \mathrm{AMMT}}$$

The collected (AMMTs) are reported in degree Celsius (°C). The minimum, maximum, and average values of the fluoride concentrations in the drinking water from various provinces of Iran are presented in Table 1. Using AMMT data, fluoride concentrations in drinking water for select Iranian provinces were calculated. They reported in Table 2.
